# Porous Organic Polymers-Supported Zeigler-Natta Catalysts for Preparing Highly Isotactic Polypropylene with Broad Molecular Weight Distribution

**DOI:** 10.3390/polym15030555

**Published:** 2023-01-21

**Authors:** Xiong Wang, Dong Wu, Xuemei Mu, Wenqian Kang, Guangquan Li, Anping Huang, Yuan Xie

**Affiliations:** Lanzhou Petrochemical Research Center, Petrochemical Research Institute, PetroChina, Lanzhou 730060, China

**Keywords:** Ziegler–Natta catalyst, porous organic polymer (POP), isotactic polypropylene, broad molecular weight distribution

## Abstract

Porous organic polymers (POPs) have attracted much attention in numerous areas, including catalysis, adsorption and separation. Herein, POP supported Ziegler–Natta catalysts were designed for preparation of isotactic polypropylene (iPP). The POPs-based Ziegler–Natta catalysts exhibited the characteristic of broad molecular weight distribution (MWD > 11) with or without adding an extra internal electron donor. The added internal electron donor 3-methyl-5-tert-butyl-1,2-phenylene dibenzoate (ID-2) used in cat-2 showed good propylene polymerization activity of 15.3 × 10^6^ g·PP/mol·Ti·h, high stereoregularity with 98.2% of isotacticity index and broad molecular weight distribution (MWD) of 12.3. Compared to the MgCl_2_-supported Ziegler–Natta catalysts (cat-4) with the same ID-2, cat-2 showed higher chain stereoregularity for propylene polymerization. As seen in the TREF results, the elution peak of PP-2 (124.0 °C, 91.7%) is 1.5 °C higher than the isotactic fraction from PP-4 (122.5 °C, 87.2%), and even 1.2 °C higher than PP-5 prepared from ID-3 with the characteristics of high stereoregularity. Moreover, the pentad methyl sequence mmmm of PP-2 (93.0%) from cat-2 is 0.5% higher than that of PP-4 from cat-4. XPS analysis revealed that the minute difference in binding energy of Ti, Mg, C and O atoms exist between the inorganic MgCl_2_ and the organic polymer based Z–N catalysts. The plausible interaction mechanism of active sites of Mg and Ti with the functional groups in the POP support and the added ID was proposed, which could be explained by their high stereoregularity and the broad molecular weight distribution of the POP-based Z–N catalysts.

## 1. Introduction

Since the discovery of the Ziegler–Natta catalyst more than 60 years ago, the consumption of polyolefins has been growing with catalyst technology and polymerization process innovations [1,2,3,4,5,6,7]. In 2021, the global consumption of polyolefins had arrived at approximately 200 million tons, and polypropylene (PP), typically isotacitc PP, is widely used in various walks of life, such as automobiles, packaging, household, medical and facilities industries [8,9], with predicted global demand of above 75 million tons in 2022.

The massive production of PP is greatly facilitated by the breakthroughs of Ziegler–Natta catalysts for high productivity and isotactic PP. Among them, the impact of the extra addition of electron donors to improve the overall performance of the supported Z–N catalysts has been distinguished. The addition of extra electron donors or Lewis bases on the MgCl_2_/TiCl_4_ surface could dramatically improve the polymerization activity, stereoselectivity and catalyst morphology, and regulate hydrogen response and molecular weight distribution by modulation of the chemical environment, content and dispersion of Ti metal sites, and the MgCl_2_ crystalline size in the supported Ziegler–Natta catalysts [10,11,12]. For recent decades, much attention has been focused on the design and fabrication of catalysts support, and on novel electron donors and their interaction mechanisms with active sites in the supported MgCl_2_ based PP catalysts [13,14,15,16,17,18,19,20,21,22].

However, the inorganic support components would eventually incorporate extra impurities and surface acidic groups in the final product, which might limit its applications in high value-added fields, such as the electronics and medical industries. As a potential clean and green alternative composed mainly of C, H and O atoms, etc., porous organic polymers (POPs) have received extensive attention in heterogeneous catalysis, including olefin polymerization, owing to their high surface area and controllable pore structure, flexible synthesis strategy and functionality [23,24,25,26,27,28,29,30]. Moreover, the less hydrophilic surface of POPs allows them to endure higher impurity contents of moisture and oxygen in the polymerization medium, need no fastidious pre-treatment, and provides a polymerization medium more close to homogeneous catalysis [31,32].

The integrated design and preparation of POP-based PP catalysts may provide a feasible and practical route for future industrial applications [33,34]. By the design and selection of functional comonomer(s), and the optimization of POP synthesis conditions, for example, the solvent solubility parameter, etc., used in a dispersion polymerization, highly flowable POP support with excellent morphology and controllable pore structure and particle size could be obtained. In addition, the POP support with designed functional groups could tune effectively the micro-chemical environment of active sites when interacted with Mg and Ti compounds. A variety of functional monomers containing hydroxyl, chloromethyl, carboxylic acid and amino group, ionic liquid, etc., have been revealed in the synthesis of POP-based polyolefin catalysts [35,36,37]. The selected functional monomer(s) would have an important influence in both the particle-forming of POP support and the modulation of the chemical environment of metal active sites, which determines the microstructure of the produced polyolefins. In our recent work, 4-vinylbenzenesulfonic acid sodium was selected as the functional comonomer in the synthesis of POP, and the sulfonated POP-based Ziegler–Natta PP catalysts exhibited excellent stereoselectivity [34], although the produced PP particles obtained relatively low bulk density due to the sulfonated POP support. Highly flowable POPs with relatively high bulk density were also reported by our group, and the POP-based metallocene catalyst exhibited excellent polymerization activities, polymer physical properties and industrialization potential [38].

In this paper, 4-hydroxyethylmethacrylate as functional comonomer was selected for integrated synthesis of highly flowable POP support with excellent physical properties, and for tailoring the micro-chemical environment as Lewis acid to interact with Ti and Mg sites. The prepared HEMA-functionalized POP-based catalysts exhibited ultrahigh stereoregularity and broad molecular weight distribution, with relatively high polymer bulk density in the propylene polymerization evaluation.

## 2. Experimental Section

### 2.1. Materials

2,2-azo-bis-isobutyronitrile (≥98%, AIBN), 4-hydroxyethylmethacrylate (≥96%, HEMA), Divinylbenzene (DVB) (80%), diisobutyl phthalate (DIBP, ID-1) and 9,9-bis(methoxylmethyl) fluorene (BMMF, ID-3) were provided from Aladdin Reagent (Shanghai, China) and pre-treated in accordance with previous literature [26,27,28]. Deionized water, propylene, triethylaluminium (TEAL, 10% in hexane solution) and methyl cyclohexyl dimethoxysilane (CHMMS, donor C) were donated by Lanzhou petrochemical company. Methylmagnesium chloride solution (3M) in tetrahydrofuran and nano titanium oxide (≥99.8%, 100 nm beads, anatase, hydrophilic) (Aladdin reagent, Shanghai, China), 3-methyl-5-tert-butyl-1,2-phenylene dibenzoate (ID-2) (Tianjin Scaxchem Limited Compnay, Tianjin, China), POE-b-POP (Poloxamer 407, BASF), ethanol (≥99.5%, SinoPharm, Beijing, China) and titanium tetrachloride were directly used without further treatment.

### 2.2. Preparation of POP Supports and POP-Based Ziegler–Natta Catalysts

In the dispersion polymerization, the thermodynamic compatibility between the solvent and the prepared polymers, the type and content of the selected functional comonomers, cross-linking degree, template agent, etc., as discussed in detail in our previous work, play a key role in the properties of the prepared POP as catalyst support [26,38]. To obtain relatively excellent properties (high SSA and bulk density, good particle flowability, etc.) as catalyst support, porous organic polymer (POP) supports were prepared according to the above mentioned references by selecting preferred solvent systems with suitable solubility parameters (ethanol/H_2_O = 9:1 volume ratio) and DVB/HEMA (3:1 molar ratio). Two samples of POP3120T (with about 20 wt% content TiO_2_, with about 1.5 mmol/g concentration of HEMA functional monomers) and POP3100 (no nano TiO_2_ added, with about 1.9 mmol/g concentration of HEMA functional monomers) were obtained and were vacuumed at 120 °C for 8 h before use.

The immobilization procedure of Ziegler–Natta catalysts was similar to our recent work [37], with some modifications. Using Schlenk techniques, 10 mL CH_3_MgCl (3M in THF solution) were added into a glass reactor containing 2.0 g POP support and 80 mL dry toluene. After reaction for about 60 min at ambient temperature, the prepared CH_3_MgCl Grignard reagent-modified POP was washed and filtered with excess toluene. After that, 20 mL TiCl_4_ was dropped slowly into the POP toluene (80 mL added ahead) solution, and stirred for 30 min. Then 0.30 g ID-1 or ID-2 was put into the reactor at 80 °C with stirring for another 1 h. After being washed in toluene and hexane, and being vacuum-dried, free-flow brown catalysts were obtained.

### 2.3. Propylene Polymerization

The immobilized Ziegler–Natta catalysts were evaluated for propylene polymerization in a 10 L autoclave. A mass of 2.0 kg propylene was added into the autoclave after nitrogen displacement, then 10 mL TEAL (10% in hexane) was introduced as scavenger and cocatalyst with stirring for 3–5 min. A mass of 100 mg of the prepared catalysts, and 0.3 mL donor C, were added, and kept the added catalyst system interaction for another 5 min. The polymerization was conducted at 70 °C with 0.3 g H_2_ for an hour. After polymerization, dried polypropylene powders could be collected.

### 2.4. Characterization

Pore structure analysis was performed on a Nova 2000e instrument (Quantachrome Instruments, Boynton Beach, FL, USA). The IR analysis was conducted on a Nicolet iS50 FT-IR (Thermo Fisher, Milwaukee, WI, USA). A Bruker D8 ADVANCE (Bruker, Karsruher, Germany) was adopted to conduct powder X-ray diffraction (PXRD) analysis using Cu Kα radiation (λ = 1.5406 Å). Ti and Mg element analyses were conducted on a VISTA ICP-MPX (Varian, Palo Alto, CA, USA) [34]. Molecular weight and its distribution was performed on a GPC-IR instrument (Polymer Char, Valencia, Spain) at 135 °C using 1,2,4-trichlorobenzene (TCB) as a solvent. Differential scanning calorimetry (DSC) analyses were tested on a DSC 214 instrument (Netzsch, Selb, Germany). Temperature rising elution fractionation (TREF) was carried out on a Polymer Char model 200+ from Spain using TCB as solvent [6]. X-ray photoelectron spectroscopy (XPS) characterization was conducted on an ESCA Lab 250 spectrometer (Thermo Fisher Scientific, Waltham, MA, USA) with Al Kα radiation at 1486.6 eV [34]. A ^13^C NMR analysis was conducted on a 500 MHz Bruker (Breika, Massachusetts, USA) at 120 °C using o-C_6_H_4_Cl_2_/o-C_6_D_4_Cl_2_ (50% *v*/*v*) as a solvent [34]. A Philips XL20 was adopted to perform the scanning electron microscope (SEM) analysis.

## 3. Results and Discussion

### 3.1. Preparation of POP Supports

**Figure 1:**
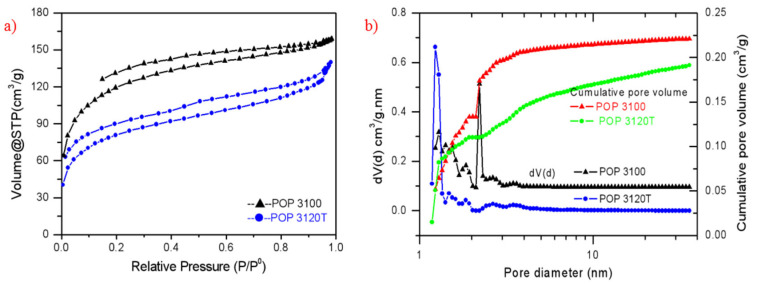
(**a**) Isotherm curves of POP3120T and POP3100; (**b**) pore size distribution curves based on nonlocal density functional theory (NLDFT) dV(d) vs. d (V: pore volume, d: pore diameter (Left); cumulative PV (Right).

Two POP supports (POP 3120T and POP 3100) were synthesized by dispersion polymerization strategy, and suitable specific surface areas (200–400 m^2^/g) for typical olefin polymerization catalyst support were obtained from both POP3120T and POP3100. As seen from Table 1, POP3100 achieved a high specific surface area (SSA) of 397 m^2^/g with a low bulk density of 0.14 g/cm^3^_,_ which will lead to low bulk density of the immobilized catalyst and the prepared polymer due to the replication effect. In order to improve the bulk density and flowability of the prepared POP support, titanium oxide nanoparticles were incorporated as a template according to our previous work [38], and a reasonable SSA of 272 m^2^/g and total pore volume (PV) of 0.216 cm^3^/g was obtained with a higher bulk density of 0.28 g/cm^3^ and excellent particle flowability. As illustrated in Figure 1, the isotherm curves of nitrogen sorption showed the porous structure of the prepared porous organic polymer, and the pore size of the prepared two POPs were mainly focused in the range from 1 to 4 nm. The average pore diameter of POP 3120T (2.478 nm) is lower than that of POP 3100 (3.172 nm), and it could be clearly observed that the highest peak (about 2.21 nm) of the pore size distribution curve of POP 3100 shifts to the left in the TiO_2_ nanoparticles based POP 3120T.

### 3.2. Preparation and Characterization of POP-Based Ziegler–Natta Catalyst

POP-based Ziegler–Natta catalysts with different internal electron donors were immobilized on POP 3120T and POP 3100 supports. As seen from Table 2, DIBP (ID-1), as a common ID, was used in cat-1, and cat-2 adopted ID-2 (3-methyl-5-tert-butyl-1,2-phenylene dibenzoate) as an internal donor, which exhibited the characteristics of high isotacticity and broad MWD typically, as could be observed in the cat-4 using inorganic spherical MgCl_2_·(xC_2_H_5_OH) support as a comparison. Cat-5 supported on the same inorganic support with cat-4 used 9,9-bis (methoxymethyl) fluorene (ID-3) as an internal donor, which has higher stereoregularity and narrower MWD for PP catalysts. No internal electron donor was adopted in cat-3. The magnesium, titanium and ID loading amounts of those prepared catalysts were analyzed and listed in Table 2. As organic supports, the Mg loading amounts of about 4.0% in the POP-based catalysts are much lower than in the inorganic supports (above 17%), while the Ti loading contents in the POP supports are similar to the organic counterparts, and the values could be controlled in the range of 3.0–4.0%. Moreover, the POP-based catalysts obtained lower ID loading contents (less than 5.0%) than the inorganic supports (about 7.0–8.0%), which could be explained by the fact that the functional group on the POPs also could interact with the metal sites as a Lewis base.

**Figure 2:**
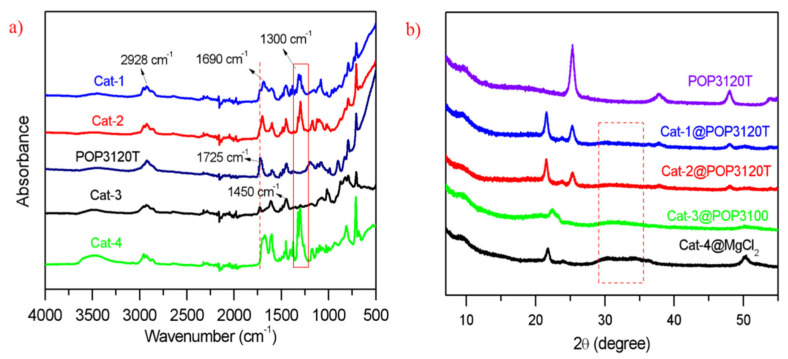
(**a**) FTIR results from the POP3120T and the prepared POP-based and MgCl_2_-based Z–N catalyst; (**b**) PXRD curves from POP3120T and the prepared catalysts containing MgCl_2_ nanocrystals. The peak around 21.7° can be assigned to the polyethylene crystal from the plastic protection film. No PE film used in POP3120T.

As illustrated in the FTIR spectra (Figure 2a), the stretching mode peaks around 2928 cm^−1^, and the in-plane bending vibration around 1450 cm^−1^ of  υ ˜(C-H) could be observed in the POP supports and all supported catalysts. The hydroxyl absorption around 3700–3200 cm^−1^ in the organic support based Ziegler–Natta catalysts are less obvious than in the inorganic support based catalyst (cat-4), despite that the hydroxyl absorption is not negligible [39]. The peaks in POP3120T and cat-3 without ID around 1725 cm^−1^ can be ascribed to the characteristic absorption of C=O in the HEMA functional units, and when internal electron donors (ID-1 and ID-2) were added into the supported catalysts, peak shifts of C=O could be observed due to the added ID-1 and ID-2 containing C=O bonds. The formed strong doublet absorption around 1700–1650 cm^−1^, can be attributed into the interaction or coordination of DIBP with Mg and Ti sites, through the oxygen atom in the C=O group. Similar vibration peaks around 1710–1660 cm^−1^ also can be observed in cat-4 with ID-2 as internal donor, which is much stronger than cat-3 with no extra ID added. The peaks around 1600 cm^−1^ are caused by the vibration of aromatic ring skeleton, which could be clearly observed in all prepared catalysts and POP3120T support. Furthermore, strong bands around 1300 cm^−1^ could be noticed in all catalysts with addition of extra ID, which is due to the C-O stretching vibration from the formed ester complexes [40]. In addition, the broad absorbance around 500 cm^−1^ can be attributed to the characteristic peak of TiO_2_ in the POP3120T support.

The PXRD analysis results of the prepared catalysts and POP3120T support are presented in Figure 2b. The diffraction peaks of TiO_2_ crystal (anatase) can be found from POP3120T support and its supported Z–N catalysts, with 2θ at 25.3° of the (101) crystal face, 37.8° of the (004) and 48.2° of the (200). The (110), (104) and (012) planes of MgCl_2_ crystal could be observed in cat-4 with 2θ around 50.5°, 35.1° and 30.5°, respectively. However, in sharp contrast, only tiny peaks appear around 30–35°, and 50.5°, indicating that fewer MgCl_2_ crystals were formed in the POP-based Z–N catalysts, partly due to significantly low Mg content. The formed broad peaks around 30–35° could be attributed into the nanosize MgCl_2_ in the catalysts [41,42], and no obvious Cl−Mg−Cl triple layers are shaped due to the lack of peaks around 15.2° in POP-based catalysts.

**Figure 3:**
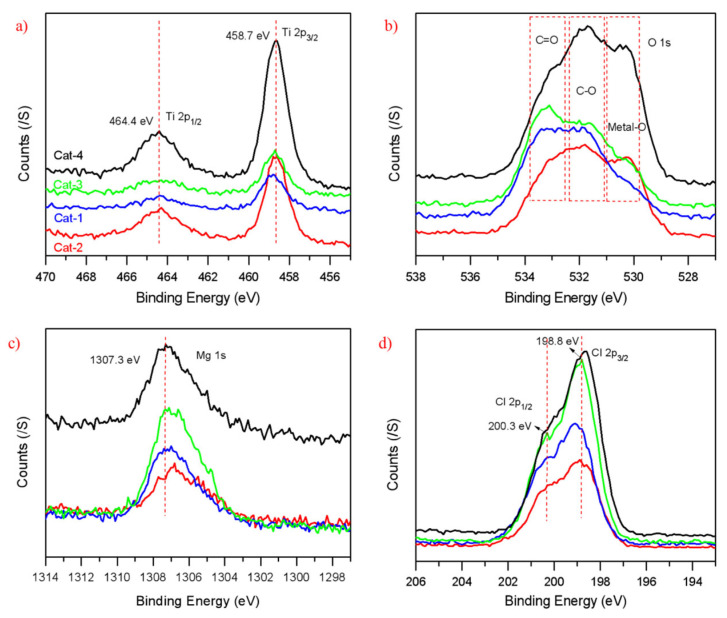
(**a**–**d**) XPS characterization results from the POP-based and MgCl_2_-based Z–N catalysts.

XPS characterization was performed to evaluate how the chemical micro-environment of metal sites (Mg and Ti) can be influenced by the interaction with the added internal donors, and the binding energy curves of Mg/Ti/Cl/O atoms are presented in Figure 3. Qualitative analysis was made in accordance with the element signals without splitting peaks because of the formed multiple bonds with each atom. Similar peak shapes of binding energy of Ti 2P_3/2_ (458.7 eV) and 2P_1/2_ (464.4 eV), with addition of the same internal donor (cat-2/cat-4), could be observed. With different interactions, with or without ID, the binding energy of Ti 2P_3/2_ of cat-1 and cat-3 change slightly from that of cat-2 and cat-4. However, the MgCl_2_-based catalyst cat-4 exhibits slightly higher Mg 1s binding energy (1307.3 eV) with stronger absorbance than other three POP-based catalysts, which might be ascribed to the higher content of Mg-Cl/Mg-O bonds formed in cat-4.

The multiple peaks of O 1s could be attributed to C=O, C-O and Ti-O/Mg-O bonds formed from the HEMA units in the POP and the added IDs, approximately around 533.8–532.5 ev, 532.5–531.0 ev and 531.0–529.5 ev, respectively. With the same internal electron, cat-2 (ID-2) obtains a similar BE curve of O 1s with that of cat-4, and the stronger BE of O 1s in cat-4 could be attributed to higher content of ID-2 and Metal-O bond. Different O 1s curves could be observed in cat-1 and cat-3 due to the different interactions with the added ID. As far as the Cl 2P is concerned, two peaks, Cl 2P_1/2_ (200.3 eV) and Cl 2P_3/2_ (198.8 eV), could be observed in cat-3 without ID, and lower BE of Cl 2P_3/2_ in cat-4 (198.6 eV) could be explained by the inorganic support nature with substantial Mg loading content. When extra ID was added in other POPs-based Z–N catalysts, a slight shift of Cl 2P to higher BE are noticed, which might be due to the withdrawing electron effect of the oxygen atom interacted with Mg and Ti atoms around Mg-Cl and Ti-Cl bonds.

**Figure 4:**
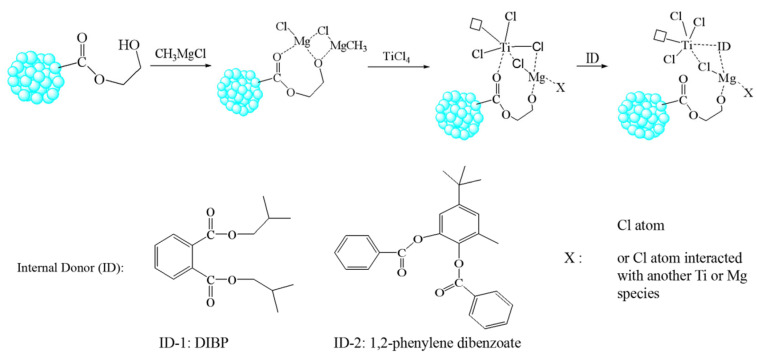
Plausible interaction mechanism of Mg/Ti active sites for the prepared POP-based Ziegler–Natta catalyst.

The plausible mechanism for how the Mg and Ti sites interact with the functional group and the ID in the POP-based Z–N catalyst was illustrated in Figure 4. Based on the loading Ti and Mg sites tethered on the HEMA functional group, no equal molar amounts of Mg or Ti atom with the HEMA functional group could be observed. In cat-2 and cat-3, the tethered Mg sites (approximately 1.7–1.8 mmol/g cat) is higher than the Ti sites (0.80–0.93 mmol/g cat) and the HEMA group (less than 1.5 mmol/g cat), therefore, extra Mg sites could be tethered around the HEMA group. In practice, the Mg/Ti molar ratio could be controlled by the added CH_3_MgCl amount or Ti/Mg molar ratio. On the POPs-based Z–N catalysts, the chemical micro-environment of Mg and Ti sites could be tuned by interacting with the functional group from HEMA monomers and the added ID thereafter. As seen from the energy dispersive spectroscopy (EDS) results in Figure S1 in the Electronic Supplementary Information, Ti, Mg elements have reacted with the POP support and the added ID containing C, O elements.

### 3.3. Propylene Polymerization by POP-Based Ziegler–Natta Catalysts

Propylene polymerization results of the prepared Z–N catalysts are provided in Table 3. As seen from Table 3, the POPs-based Z–N catalysts exhibited broad MWD (11–17) with different internal electron donors (ID-1 and ID-2), or even without adding extra internal electron donors. Typically, the added ID could play a vital role in the supported Z–N catalysts, and it might influence dramatically their properties, including propylene polymerization activity, molecule chain stereoregularity and hydrogen response, etc. As a comparison, the MgCl_2_-based Z–N catalysts with DIBP as ID have a relatively narrower MWD (6–8), while 3-methyl-5-tert-butyl-1,2-phenylene dibenzoate as ID has a broad MWD, as cat-4 obtained a broad MWD of 11.0. In addition, the broad MWD iPP could obtain better balanced mechanical and processing performance than common iPP. It could be well explained that the HEMA functional group in the POPs can modulate effectively the chemical micro-environment of the immobilized Ti and Mg active sites after interaction with CH_3_MgCl and TiCl_4_, and the broad MWD of the prepared PPs could be ascribed partly to the HEMA functionalized POP support.

**Figure 5:**
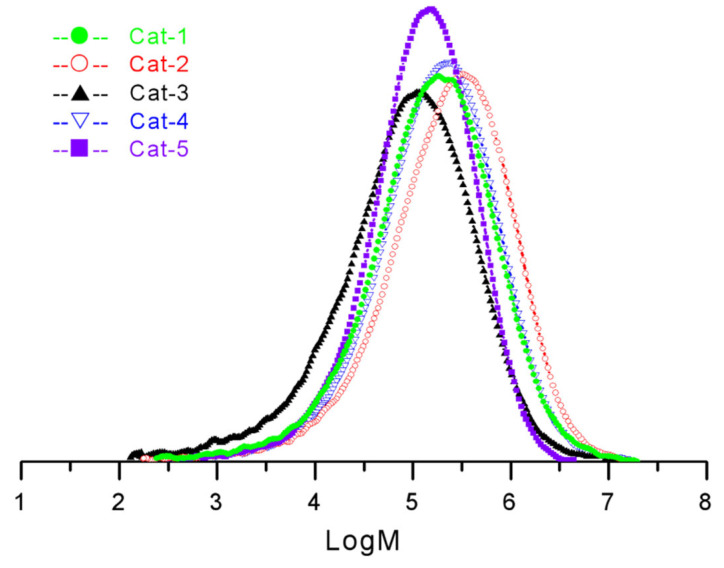
Molecular weight distribution curves of the prepared PPs from five different catalysts.

Furthermore, propylene polymerization activity and its chain stereoregularity could be improved apparently by addition of ID. Without the addition of ID, cat-3 obtains relatively low propylene polymerization activity of 4.3 × 10^6^ g·PP/mol·Ti·h with a low isotactic index of 91.5%, which caused obvious reactor fouling after polymerization. Upon addition of extra ID-1 and ID-2, the polymerization activities of the prepared POP-based Z–N catalysts cat-1 and cat-2 increased remarkably to 10.5 × 10^6^ g·PP/mol·Ti·h and 15.3 × 10^6^ g·PP/mol·Ti·h, respectively, and the molecular chain stereoregularity also increased with isotactic index from 91.5% to 97.5% and 98.2%. Interestingly, cat-2 obtained higher stereoregularity than cat-4 with the same internal electron donor (ID-2), and the TREF, ^13^CNMR analysis results discussed later could also confirm it. Although the MgCl_2_-based Z–N catalyst with ID-3 (cat-5) achieved higher chain stereoregularity with isotactic index of 98.4%, only a narrow MWD of 5.3 is gained. The molecular weight distribution curves of the prepared PPs from five different catalysts were shown in Figure 5, and the polymer prepared from cat-5 has an evidently narrower molecular weight distribution than those polymers prepared from other four catalysts.

**Figure 6:**
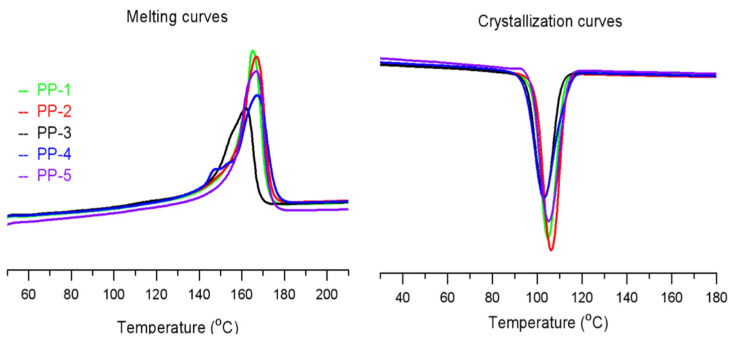
DSC results of the PPs obtained from the POP-based and MgCl_2_-based Z–N catalysts.

DSC, TREF and ^13^CNMR analyses were characterized to further evaluate the microstructure of PP molecular chains. As discussed above, a high isotacticity index (≥97%) could be obtained from the Z–N PP catalysts with extra IDs from Soxhlet extraction method using n-heptane, and the stereoregularity of PP molecular chains is highly related to their melting and crystallization behaviors. From Figure 6 and Table 4, the PP melting peaks Tm from the prepared catalysts vary from 161.9 °C to 167.2 °C. PP-3, with low isotacticity index from cat-3, obtains the lowest melting peak of 161.9 °C and melting enthalpy of 86.1 J/g, and PP-4 from cat-4 gains the highest Tm value of 167.2 °C, while with a relatively low melting enthalpy of 103.0 J/g. Using the same ID-2, PP-2 from cat-2 also obtains a higher Tm of 166.8 °C and enthalpy of 110.8 °C, which is close to PP-5 from cat-5 with the highest isotacticity index. The melting enthalpy is in good accordance with the isotacicity index of PPs. In addition, the higher crystalline peak Tc of PP-2 from POP-based catalysts cat-2 could be observed, indicating faster crystalline growth.

**Figure 7:**
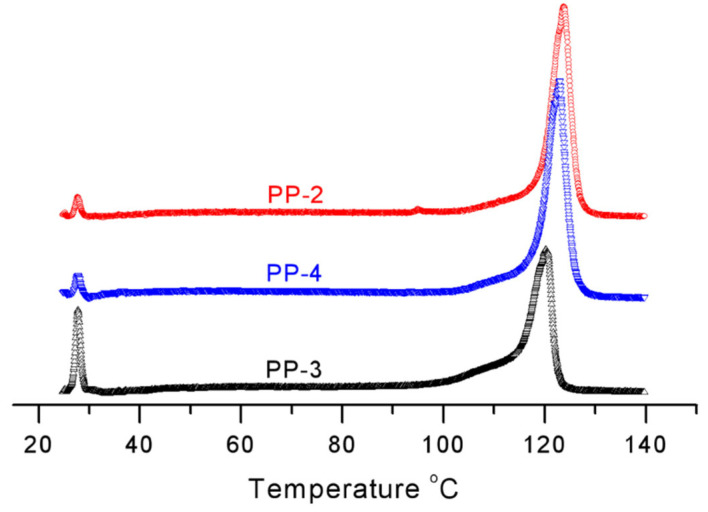
TREF results of the obtained PPs from cat-2/3/4.

The isotactic sequence and its distribution could also be evaluated by the TREF and ^13^CNMR analysis. As for the homo-polypropylene, the soluble fractions (SF) can be ascribed to the atactic PP chains, and the results are in good agreement with the isotacticity index from n-heptane extraction. The highest soluble fraction of 8.4% is corresponding to PP-3 (i.i.: 91.5%), prepared from cat-3 without ID, and PP-1 (i.i.: 97.5%), from cat-1 with ID-1, gained low SF of 2.7% (see Table 5). In contrast, with the same ID-2, PP-2 and PP-4 obtain different SF fractions of 1.9% and 3.2%, respectively, and the obvious decrease in the atactic component from PP-2 might be caused by the different chemical environment around the POP support. The lowest SF of 1.6% is obtained by PP-5, prepared from ID-3, which typically has high stereoregularity of PP chain. When increasing the elution temperature, the elution fractions match decreased defects in the crystalline PP chains with increased stereoregularity or more isotactic methyl sequences. The elution fractions with peak temperature around 122 °C correspond to the highly isotactic PP chains, and for the commercial iPPs (i.i.: 96–99%), the elution temperatures typically range from 121 to 123 °C with increased isotactic sequence. As seen from Table 5 and Figure 7, the added ID-1 and ID-2 can dramatically increase the stereoregularity of PP chains compared to cat-3 without ID, and the elution peak of PP-1 raises up to 121.6 °C from 119.9 °C, and PP-2 increases to 124.0 °C. The MgCl_2_ based Z–N catalysts cat-4 and cat-5 exhibit high stereoselective ability with the elution temperature of 122.5 °C (peak area: 87.2%) from PP-4 and 122.8 °C (94.0%) from PP-5. In sharp contrast, the elution peak of PP-2 is even 1.5 °C higher than that of PP-4, prepared from the same ID-2, with a higher fraction of 91.7%, and 1.2 °C higher than PP-5, prepared from ID-3, with the characteristics of high stereoregularity. As a result, it could be well-reasoned that the POP-based Z–N catalyst with ID-2 has ultrahigh stereoregularity ability on the growing chains. The minor peaks with relatively low contents are due to fewer isotactic sequences existing in their PP chains, as provided by the built-in model from the TREF analysis.

**Figure 8:**
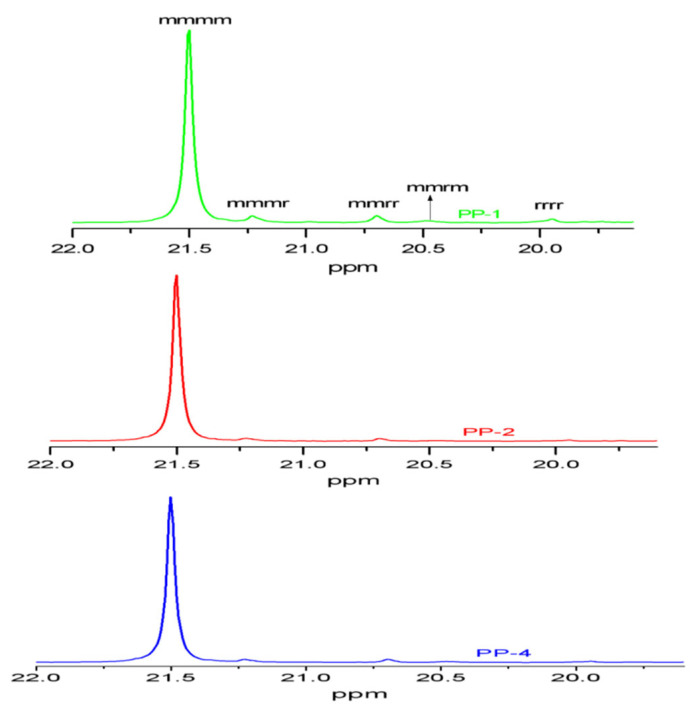
The ^13^CNMR spectra of three PP samples from cat-1, cat-2 and cat-4.

On the basis of ^13^CNMR analysis, sequence distributions of methyl can be determined by its chemical shift from about 19.5 ppm to 22.0 ppm, and the calculation method of pentad sequence distribution is given in the supplemental information. From Figure 8, 5 pentad sequences, including mmmm (around 21.5 ppm), mmmr (21.2 ppm), mmrr (20.7 ppm), mrmm (20.4 ppm) and rrrr (19.9 ppm) sequences, could be identified, and the calculation results of methyl sequence of PP-1/PP-2/PP-4 are presented in Table 6. PP-2 obtained more isotactic mmmm sequences and fewer atactic rrrr sequences than PP-4 and PP-1, which is in good agreement with previous analysis.

### 3.4. Surface Morphology of Prepared PP

**Figure 9:**
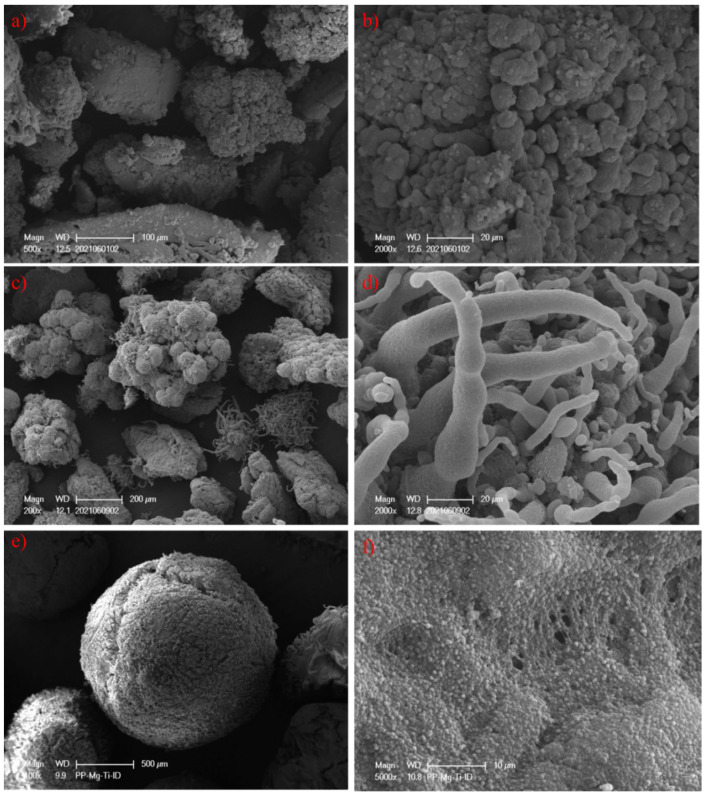
SEM photos of PP-1 from cat-1 (**a**,**b**); PP-2 from cat-2 (**c**,**d**); PP-4 from cat-4 (**e**,**f**).

The particle morphology of PP-1, PP-2 and PP-4 is illustrated by scanning electron microscopy (SEM), and the surface morphology of POP3120T is shown in Figure S2 (see Supplemental Information). As shown in Figure 9, the particle morphology of PP-1 and PP-2 prepared from POP-based PP catalysts (cat-1 and cat-2) differs from the spherical MgCl_2_ based catalyst (cat-4). Typically, by replication effects, the PP-4 particles prepared from the spherical catalyst obtain similar shapes as the polymer grows in the MgCl_2_ support, and fragmentation might happen on the sphere surface with cracks due to the exothermic polymerization. PP-1 and PP-2 particles take on irregular granule appearances, which are originated from aggregates of smaller POP particles with similar shapes. Surprisingly, apart from the irregular granules, some strings attached on the granules could be observed in PP-2, which might be caused by gaps or space on the surface, and the polymerized fibers at the initial stage grows further to form strings around the gaps.

## 4. Conclusions

Porous organic polymer (POP)-based Ziegler–Natta catalysts were developed for propylene polymerization, and the prepared catalysts with the selected internal electron donor obtained ultrahigh stereoregularity and broad molecular weight distribution. By the integrated design of POP-based PP catalysts, the selected functional comonomer HEMA play a significant role, not only in the support forming, but also in the modulation of the chemical micro-environment of Ti, Mg sites, with further interaction with the chosen internal donor. This integrated design of the POP-based catalyst provides a flexible method to modify the metal active sites for development of specialty polyolefin, which would be practicable for future commercialization of novel and high value-added polyolefins, and a beneficial supplement to the inorganic MgCl_2_-based Z–N catalysts.

## Figures and Tables

**Table 1:** Porosimetry analysis from N_2_ sorption and bulk density results.

Support	Specific Surface Area m^2^/g	Total Pore Volume cm^3^/g	Average Pore Diameter nm	Bulk Density g/cm^3^	Particle Flowability
POP3120T	272	0.216	3.172	0.28	excellent
POP3100	397	0.246	2.478	0.14	good

Note: POP3120T means that the support was prepared with about DVB/HEMA = 3:1 (molar ratio) and 20% TiO_2_ weight content in the final POP support; POP3100 means that the support was prepared with about DVB/HEMA = 3:1 without adding TiO_2_.

**Table 2:** Supported Ziegler–Natta catalysts for propylene polymerization.

Catalyst No.	Support	Internal Electron Donor (ID)	Mg (wt%)	Ti Loading Amounts (wt%)	ID (%)
Cat-1	POP3120T	ID-1	4.0	4.0	4.8
Cat-2	POP3120T	ID-2	4.2	3.5	4.2
Cat-3	POP3100	No ID	3.9	3.2	-
Cat-4	MgCl_2_	ID-2	17.8	3.2	7.1
Cat-5	MgCl_2_	ID-3	17.2	3.0	7.6

**Table 3:** Propylene polymerization evaluation results from the prepared POP-based and MgCl_2_-based Z–N catalysts.

Catalysts No.	Activity (×10^6^ g·PP/mol·Ti·h)	Productivity (kg·pp/g·cat·h)	Bulk Density (g/mL)	Isotactic Index (%)	Mw	Mn	Mw/Mn
Cat-1	10.5	8.8	0.33	97.5	39.40	3.47	11.4
Cat-2	15.3	11.2	0.31	98.2	55.06	4.46	12.3
Cat-3	4.3	2.5	0.24	91.5	23.45	1.44	16.3
Cat-4	54.5	36.4	0.40	97.0	41.45	3.77	11.0
Cat-5	39.1	24.5	0.38	98.4	22.91	4.33	5.3

**Table 4:** DSC results.

Sample	*T*_c_ (Peak) °C	*T*_c_ (Onset) °C	*T*_m_ (Peak) °C	Δ*H*_m_ J/g
PP-1	104.9	111.9	165.0	105.3
PP-2	106.3	113.0	166.8	110.8
PP-3	103.3	110.5	161.9	86.1
PP-4	103.0	113.3	167.2	103.0
PP-5	105.2	113.6	166.5	113.9

**Table 5:** TREF results.

Sample	Item	Soluble Fraction (SF)	Peak 1	Peak 2	Peak 3	Peak 4
PP-1	T/^o^C		51.2	70	84.4	121.6
	Area/%	2.7	3.1	1.9	1.4	90.8
PP-2	T/^o^C		47.1	55.5	80.1	124.0
	Area/%	1.9	2.7	2.2	1.8	91.7
PP-3	T/^o^C		51.1	57.6	81.2	119.9
	Area/%	8.4	3.0	2.2	2.4	84
PP-4	T/^o^C		48.2	52.7	59	122.5
	Area/%	3.2	4.5	2.0	3.1	87.2
PP-5	T/^o^C		60.0	72.9	75.4	122.8
	Area/%	1.6	1.3	1.0	2.1	94.0

**Table 6:** Methyl pentad sequence distribution results of iPP from ^13^C NMR analysis.

Sample No.	Methyl Pentad Sequence Distribution (%)								
	mmmm	mmmr	rmmr	mmrr	mrmm	mrmr	rrrr	rrrm	mrrm
PP-1	87.3	4.68	0	3.81	1.83	0	2.38	0	0
PP-2	93.0	3.17	0	1.96	0.83	0	1.04	0	0
PP-4	92.5	3.07	0	2.19	1.13	0	1.11	0	0

## Data Availability

Not applicable.

## Supplementary Materials

The following supporting information can be downloaded at: https://www.mdpi.com/article/10.3390/polym15030555/s1, Figure S1: Energy dispersive spectroscopy (EDS) analysis results of POP and prepared Cat-1/2/3/4; Figure S2: SEM photos of POP3120T support.
