# Supplementary material for: Porous Organic Polymers-Supported Zeigler-Natta Catalysts for Preparing Highly Isotactic Polypropylene with Broad Molecular Weight Distribution
Source: Polymers (Basel). 2023 Jan 21;15(3):555. doi: 10.3390/polym15030555 (PMC9919294; doi:10.3390/polym15030555)
Supplement: Supplementary file 1 [file polymers-15-00555-s001.zip › polymers-2098683-supplementary.pdf]

Supplementary Material: Porous organic polymers-supported Zeigler-Natta catalysts for preparing highly isotactic polypropylene with broad molecular weight distribution

Xiong Wang, Dong Wu, Xuemei Mu, Wenqian Kang, Guangquan Li, Anping Huang and Yuan Xie

Table of contents

1. Figure S1. Energy dispersive spectroscopy (EDS) analysis results of POP and prepared Cat-1/2/3/4.
2. Figure S2. SEM photos of POP3120T support. The SEM characterization of POP support was conducted on a HITACHI S4800 (Tokyo, Japan).

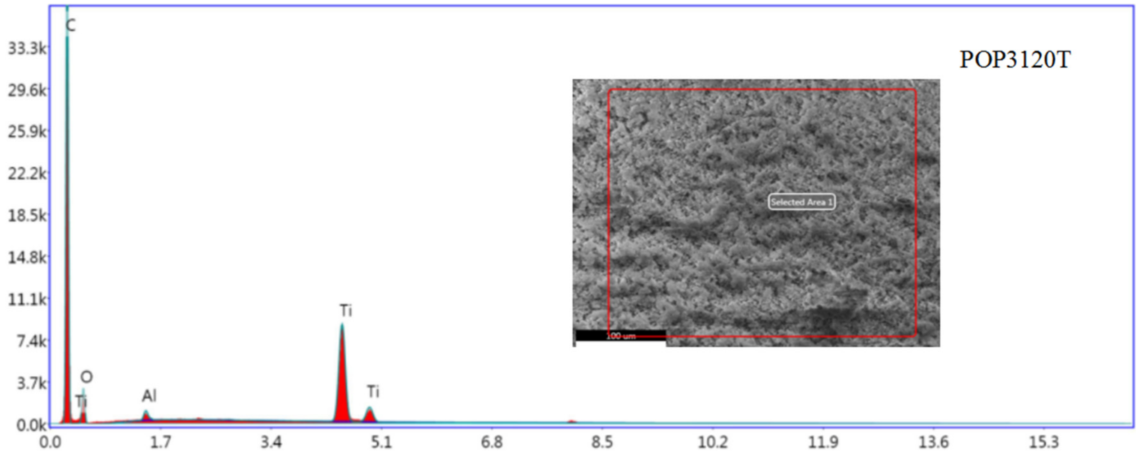

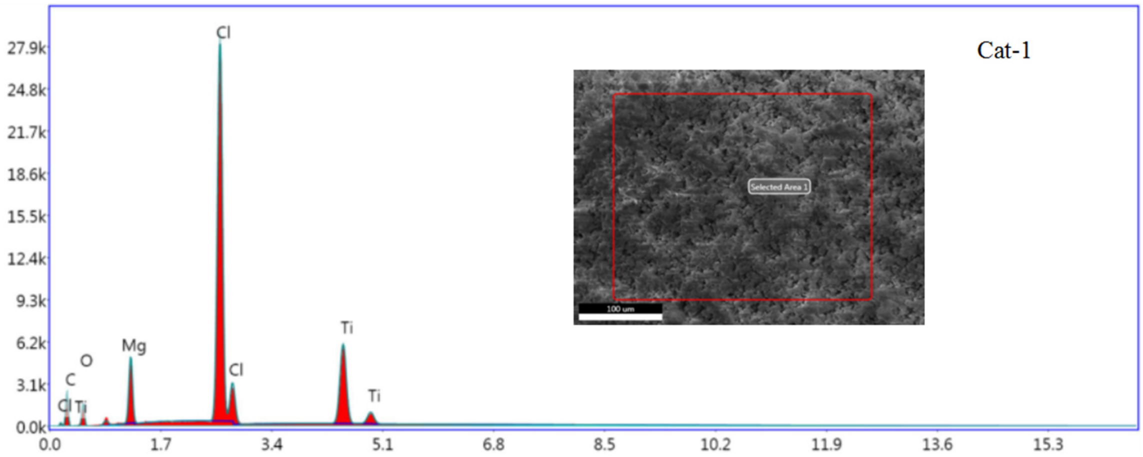

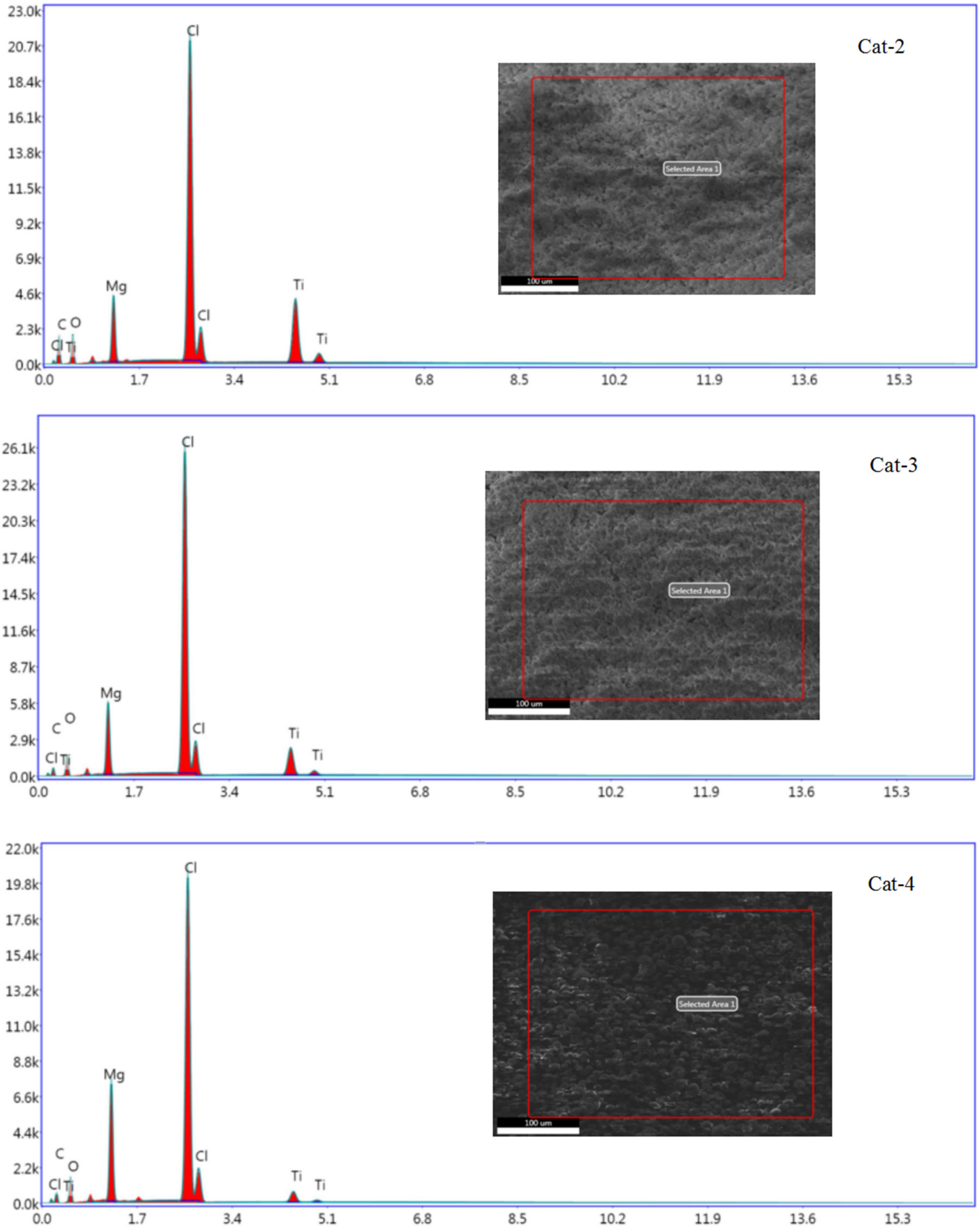

Figure S1. Energy dispersive spectroscopy (EDS) analysis results of POP3120T and prepared Cat-1/2/3/4. EDS mapping was conducted on a Team Octane Prime (EDAX, USA) equipped on a Philips XL20 SEM.

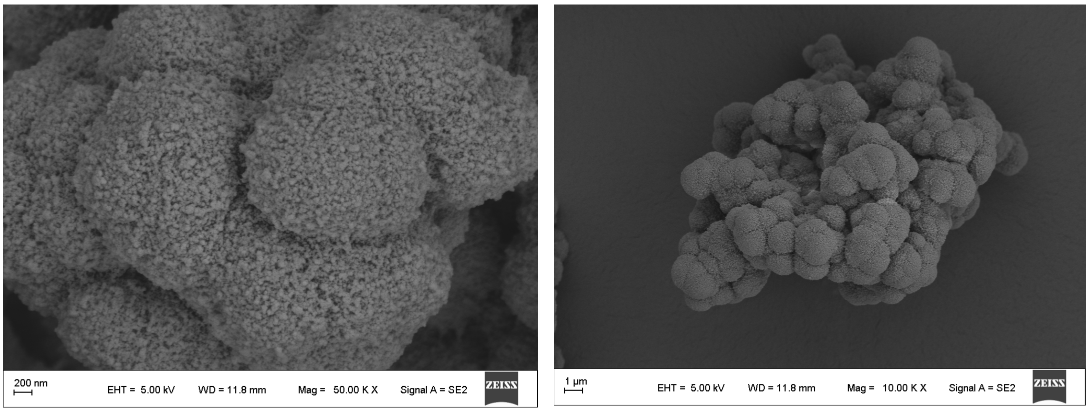

Figure S2. SEM photos of POP3120T support.

Calculation method of mmmm sequence:

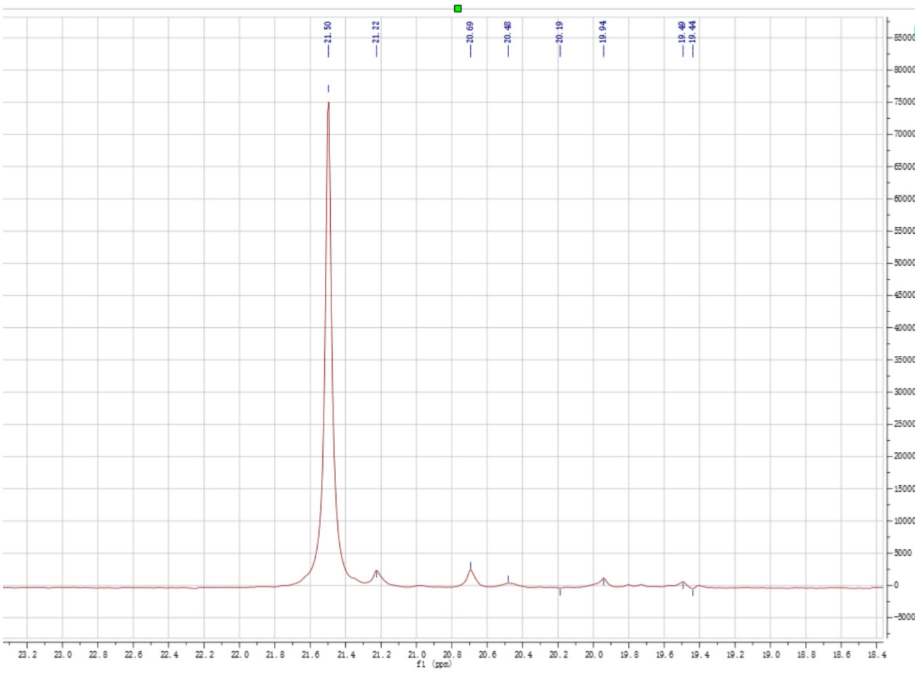

In this case, 5 signal peaks were observed, with [mmmm] , [mmmr], [mmrr], [mmrm], [rrrr] from left to right. The last peak is caused by S/N ratio. The isotacticity was caculated by [mmmm]/([mmmm]+ [mmmr]+ [mmrr]+ [mmrm]+ [rrrr]). (peak area value)
